# A Rare MPIG6B Gene Mutation in a Saudi Adolescent Male With Thrombocytopenia, Anemia, and Myelofibrosis: A Case Report

**DOI:** 10.7759/cureus.54042

**Published:** 2024-02-11

**Authors:** Badriah G Alasmari, Mohammed Alpakra, Sara Saeed, Syed Rayees, Lina Elzubair, Abrar Aljunaid

**Affiliations:** 1 Department of Pediatrics, Armed Forces Hospital Southern Region, Khamis Mushait, SAU; 2 Department of Hematology and Oncology, Armed Forces Hospital Southern Region, Khamis Mushait, SAU; 3 Department of Pathology, Armed Forces Hospital Southern Region, Khamis Mushait, SAU; 4 Department of Pediatrics, International Medical Center, Jeddah, SAU

**Keywords:** myelofibrosis, mpig6b, thamy, hematology, pediatrics

## Abstract

Thrombocytopenia, anemia, and myelofibrosis (THAMY) is an exceptionally rare autosomal recessive inherited disorder that arises from pathogenic variations in the megakaryocyte platelet inhibitor G6B (MPIG6B) gene. The MPIG6B gene plays a crucial role in regulating platelet homeostasis. The hallmarks of THAMY are macrothrombocytopenia and focal myelofibrosis, accompanied by varying degrees of anemia, leukocytosis, splenomegaly, and a mild to moderate propensity to bleed. In this case report, we present the clinical details of a 13-year-old male who displayed symptoms of anemia and bleeding as a result of thrombocytopenia. Analysis of the peripheral blood smear revealed the presence of macrothrombocytes, while physical examination showed splenomegaly. To delve deeper into the matter, a bone marrow biopsy was conducted, which unequivocally confirmed the existence of focal myelofibrosis. Subsequent genetic analysis validated the homozygous variant c.523C>T in the MPIG6B gene.

## Introduction

Congenital or inherited myelofibrosis is an extremely rare occurrence, with less than 20 reported cases worldwide. Interestingly, all of these cases have been identified in consanguineous families, suggesting an autosomal recessive mode of inheritance [1]. On the other hand, myelofibrosis is commonly observed in individuals belonging to the adult age group. Primary myelofibrosis in adults is a chronic myeloproliferative disorder that arises due to somatic mutations and is not inherited. These mutations primarily affect the *JAK2*, *CALR*, or *MPL* genes [2]. Additionally, the megakaryocyte platelet inhibitor G6B (MPIG6B) gene, which encodes the G6B protein, plays a crucial role in regulating platelet production and function. The deficiency of G6B has been identified as a potential cause of thrombocytopenia, myelofibrosis, and anemia in both human and murine populations [3].

## Case presentation

**Figure 1:**
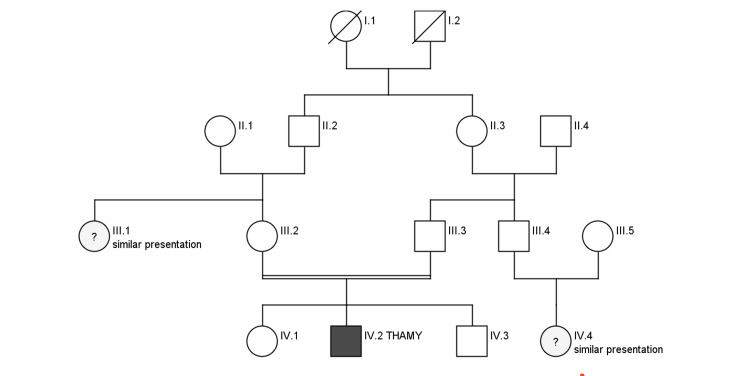
Family pedigree III.1 and IV.4 had similar presentations to our patient but they did not get the genetic testing done. THAMY: thrombocytopenia, anemia, and myelofibrosis

We present a case study of a 13-year-old Saudi male born to consanguineous parents. There was a family history of a bleeding disorder in his maternal aunt, although his parents were unaware of the specific disease she had (Figure 1). The patient presented to the emergency department with complaints of epistaxis, fever, lethargy, and dizziness. He had been experiencing intermittent episodes of epistaxis for a year, but his parents did not seek any medical advice during that time. Upon physical examination, the patient looked pale, showed no dysmorphic features, and appeared to be developmentally normal. His chest was clear, the cardiovascular system was normal with regular heart sounds, and his abdomen was soft and non-tender.

**Figure 2:**
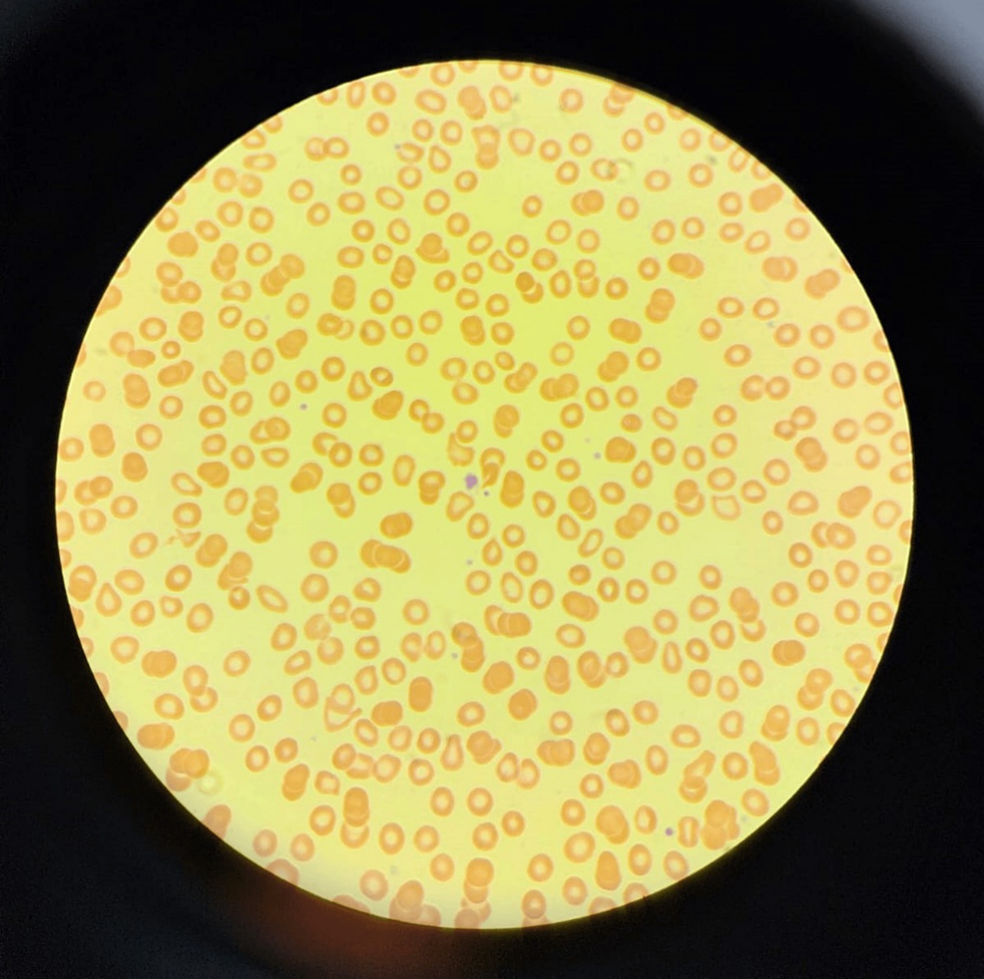
Peripheral blood smear (Wright-Giemsa stain) showing hypochromic microcytic red blood cells +2, anisopoikilocytosis +1, moderate thrombocytopenia with occasional large-sized platelets, no platelet clumps and manual platelets count of 80x10^9/L

Initial investigations comprised a complete blood count, revealing anemia with moderate thrombocytopenia. The iron profile was tested, and the results were suggestive of iron deficiency anemia (Table 1). A nasal swab for COVID-19 came back positive. The peripheral blood smear analysis showed anisopoikilocytosis and occasional large platelets (Figure 2). However, other tests such as the coagulation profile, liver function test, high-performance liquid chromatography, reticulocyte count, vitamin D, vitamin B12, and folate were all within normal ranges.

**Table 1:** Complete blood count and iron profile

Measured entity	Value on admission	Normal range
Hemoglobin	6.2 g/dl	10.9-15 g/dl
Platelets	65 x 10^9^/L	150-400 x 10^9^/L
White blood cell	4.5 x 10^9^/L	4.5-13.5 x 10^9^/L
Mean corpuscular volume	53.9 fl	76-96 fl
Ferritin	3.70 µg/L	10.3-55.8 µg/L
Iron	4.00 μmol/L	12.5-32.2 μmol/L
Unsaturated iron-binding capacity	62.60 μmol/L	27.7-63.6 μmol/L
Total iron-binding capacity	66.60 μmol/L	(reference range not available)
Transferrin saturation	6.01	<50%

In light of the symptomatic anemia, the patient received a transfusion of packed red blood cells at a dosage of 10 ml/kg. His condition was closely monitored, and he was treated symptomatically. A repeat complete blood count showed a hemoglobin level of 8.2 g/dl and a platelet count of 80 x 10^9^/L. The diagnosis at discharge was iron deficiency anemia and thrombocytopenia caused by COVID-19 infection. The patient was discharged home on iron supplements with a follow-up appointment scheduled at the hematology clinic.

**Figure 3:**
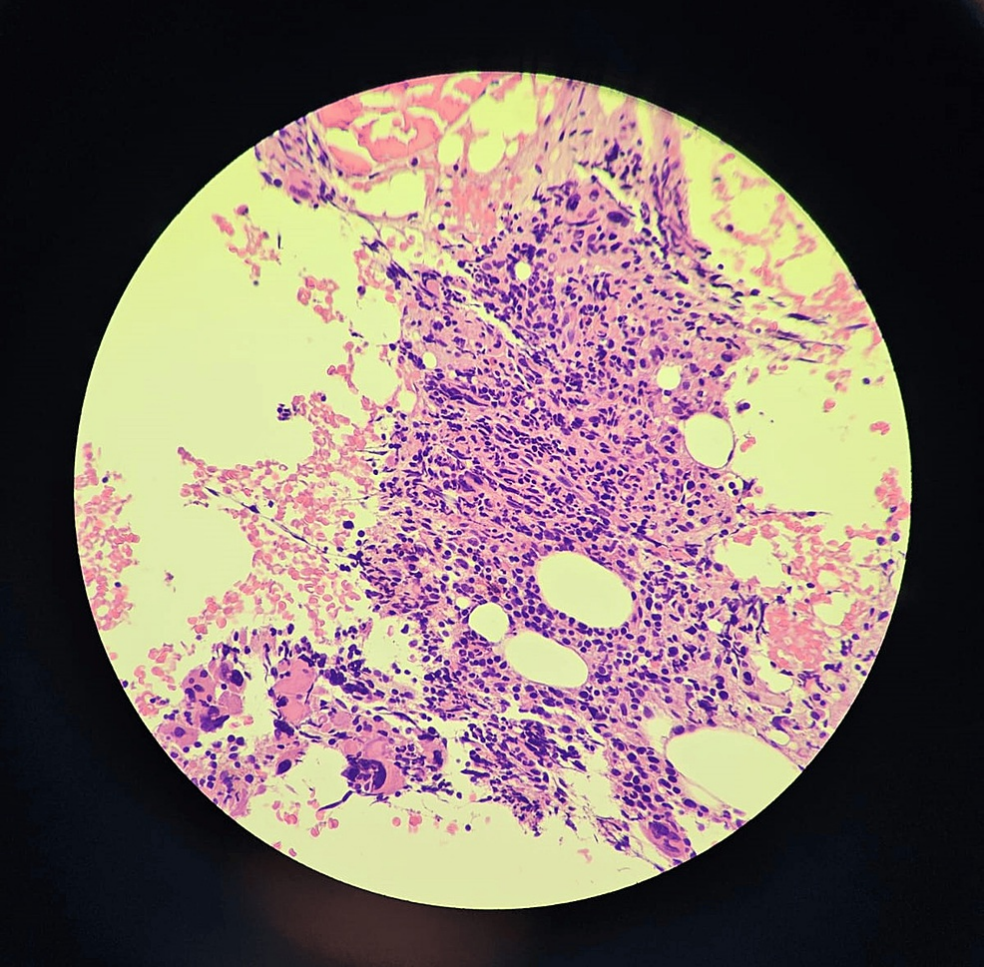
Trephine bone marrow biopsy with H&E stain (40x magnification) showing focal fibrosis and megakaryocytic hyperplasia with some atypical forms

**Figure 4:**
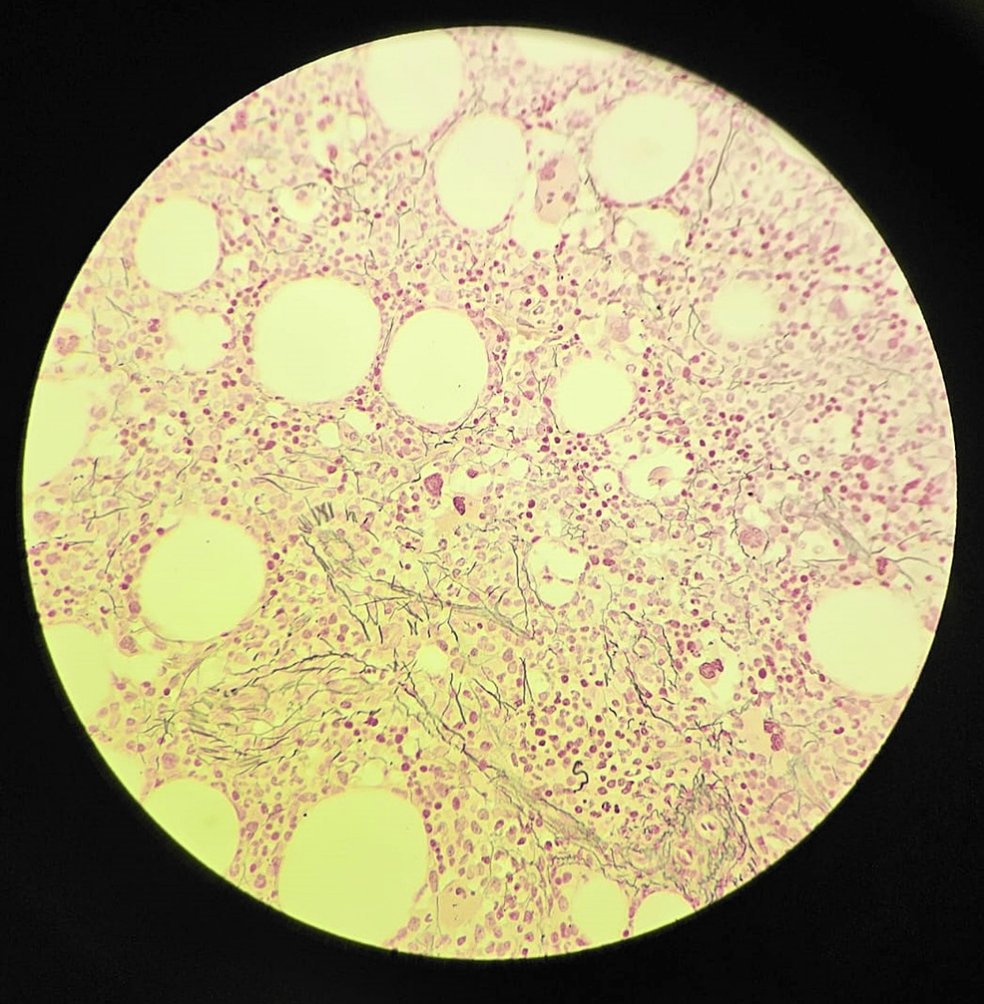
Trephine bone marrow biopsy (40x magnification with Reticulin stain) showing focal grade I-II fibrosis

**Figure 5:**
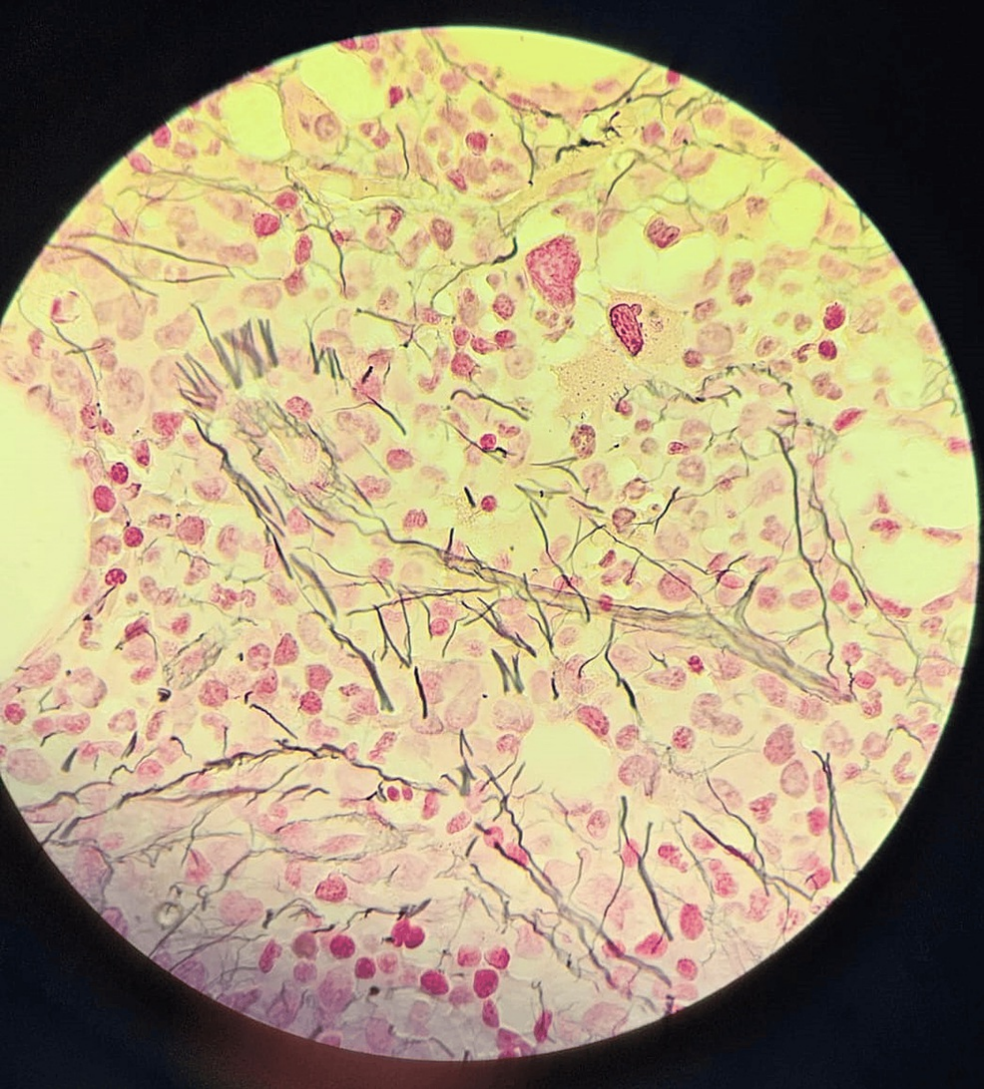
Trephine bone marrow biopsy (100x magnification with Reticulin stain) showing focal grade II fibrosis

During the follow-up course in the clinic, the patient continued to experience persistent anemia and thrombocytopenia. On physical examination, he looked pale, lethargic, and the tip of his spleen was palpable in the left hypochondrium. An ultrasound of the abdomen and pelvis revealed an enlarged spleen with a span measuring 125 mm and a homogenous echo pattern. No other abnormalities were found. Due to the patient's family history and persistent bicytopenia, additional investigations were conducted, which included a bone marrow biopsy and a whole exome sequencing study (WES). The bone marrow biopsy revealed focal fibrosis with megakaryocytic hyperplasia (Figures 3-5). The WES study identified a homozygous variant c.523C>T in the MPIG6B gene (Table 2). This finding aligns with the confirmed diagnosis of thrombocytopenia, anemia, and myelofibrosis (THAMY).

**Table 2:** Whole exome sequencing study MIM: Mendelian Inheritance in Man, AR: Autosomal Recessive, gnomAD: genome Aggregation Database, MAF: Minor Allele Frequency

Gene (isoform)	Phenotype MIM number (Mode of Inheritance)	Variant	Zygosity	MAF gnomAD (%)	Classification
MPIG6B (NM_025260.3)	617441 (AR)	c.523C>T p.(Arg175*) chr6:31692386	Homozygous	0	Pathogenic

The patient’s clinical course included controlled episodes of epistaxis. He received three packed red blood cell transfusions at 10 ml/kg and one platelet transfusion at 10 ml/kg. Recent laboratory investigations as of November 2023 showed a hemoglobin level of 10.5 g/dl and platelets of 85 x 10^9^/L. Given his stable condition, the patient is not considered a candidate for hematopoietic stem cell transplantation currently.

The patient and his parents received counseling regarding the medical condition. They were informed about the significance of regular follow-up appointments, as well as the potential necessity for a bone marrow transplant in the future.

## Discussion

Congenital macrothrombocytopenia with focal myelofibrosis is a rare autosomal recessive disease that is linked to germline ‘loss of function’ mutations in G6b (also known as G6b-B or MPIG6B). The occurrence of primary myelofibrosis in children has been linked to mutations in* MPL, VPS45, *and* RBSN* [3]. Identification of the MPIG6B gene as a cause of myelofibrosis in children has only recently been observed. At present, the scarcity of literature and cases hinders the ability to ascertain whether these germline mutations elevate the likelihood of developing a myeloid neoplasm [4]. The gene MPIG6B, alternatively referred to as G6B or C6orf25, is situated within the class III region of the major histocompatibility complex. It is specifically expressed on platelets and plays a crucial role in the differentiation of red blood cells and platelets. Myelofibrosis, a condition characterized by the abnormal growth of fibrous tissue in the bone marrow, can be attributed to various mechanisms. In cases where the G6B gene is mutated, one probable mechanism leading to myelofibrosis involves dysplastic megakaryocytes. These abnormal cells release cytokines, such as transforming growth factor-β, which contribute to the development of myelofibrosis [5]. Additionally, an autoimmune process can also play a role in the pathogenesis of myelofibrosis. Persistent inflammation triggered by this autoimmune response can lead to the development of myelofibrosis in the bone marrow. It is worth noting that mutated G6B is expressed in CD4+ T-cells and may contribute to immune dysregulation [5]. Inflammation is more commonly observed in the bone marrow, suggesting a potential involvement of inflammation or immune dysregulation in the development of myelofibrosis in these cases. Patients who have disease-causing mutations in the MPIG6B gene exhibit symptoms such as congenital macrothrombocytopenia, mild-to-moderate bleeding tendencies, focal myelofibrosis, and atypical megakaryocytes. Nevertheless, the precise etiology of this condition has yet to be fully elucidated [6]. Abnormal cell division or differentiation can affect the progenitor cells in the bone marrow, leading to blood anomalies like anemia and thrombocytopenia [7]. Thrombocytopenia is defined by low platelets below 150,000/microliter and anemia is defined by low hemoglobin levels below 11 g/dl.

The majority of the reported cases are of Arab descent, one of European descent, one of Chinese descent, and one of Indian descent. Males are affected more than females [4,5]. Several pathogenic MPIG6B mutations have been reported in 11 males and seven females with different variant mutations in the MPIG6B gene; c523C>T has been found in only one case to date [5]. Clinically, it manifests with bleeding, bruising, thrombocytopenia, and anemia in early childhood. Splenomegaly and bone marrow fibrosis eventually develop and worsen over time [4].

WES study and bone marrow biopsy are instrumental in the identification and establishment of the diagnosis of THAMY. Disease management strategies for individuals with germline G6B mutations typically encompass a range of treatment options such as red blood cell transfusions, corticosteroids, intravenous immunoglobulin, splenectomy, and hematopoietic stem cell transplant. While corticosteroids and splenectomy may provide temporary relief in certain instances, hematopoietic stem cell transplants have the potential to offer a curative outcome for a select section of patients [4].

## Conclusions

MPIG6B mutation is an extremely uncommon condition. The presence of significant consanguinity increases the likelihood of developing such rare genetic disorders. The occurrence of early-onset anemia accompanied by persistent megathrombocytopenia should raise suspicion for the presence of these rare inherited conditions. It is imperative to diligently observe blood counts to expeditiously identify and proficiently handle these patients. Families afflicted by these conditions require comprehensive and appropriate genetic counseling to fully understand the implications and potential risks involved.
